# Optical coherence tomography patterns and outcomes of contusion maculopathy caused by impact of sporting equipment

**DOI:** 10.1186/s12886-018-0843-x

**Published:** 2018-07-16

**Authors:** Danjie Li, Hideo Akiyama, Shoji Kishi

**Affiliations:** 1 Department of Ophthalmology, Gunma University School of Medicine, 3-39-15 Showa-machi, Maebashi, Gunma 371-8511 Japan; 2 Aier eye hospital (Cheng Du), 115 Xiyiduan, Yihuanlu,, Chengdu, 610041 China; 3 Maebashi Central Eye Clinic, Maebashi, Gunma Japan

**Keywords:** Optical coherence tomography (OCT), Contusion maculopathy, Commotio retinae, Incomplete macular hole, Full-thickness macular hole, Foveal hemorrhage

## Abstract

**Background:**

To describe the patterns and outcomes of contusion maculopathy after ocular contusions resulting from accidental impact with sporting equipment.

**Methods:**

We conducted a retrospective study of interventional case series. Patient Population: Twenty-one eyes of 21 patients who sustained blunt ocular trauma while playing a sport. Intervention/Observation Procedure(s): Surgery or observation by optical coherence tomography (OCT). Main Outcome Measure(s): The morphologic changes within the macula in the early stages after injury and changes in visual function in the early and recovery stages after injury.

**Results:**

In the early stage, OCT visualized four injury patterns: type Ι, commotio retinae (14.3%, 3 eyes) with increased reflectivity of the ellipsoid zone and retinal pigment epithelium; type II, incomplete macular hole(38.1%, 8 eyes) with three structural changes, i.e., a partial V-shaped macular hole, a jar-shaped macular hole with retinal tissue at the bottom, and a connective bridge attached to retinal tissues; type III, full-thickness macular hole (33.3%, 7 eyes); and type IV, foveal hemorrhage (14.3%, 3 eyes). During recovery, OCT images of types Ι and II showed almost normal macular morphology with better visual acuity (mean ± SD,0.02 ± 0.1 and 0.14 ± 0.21logMAR.). In types III and IV, the visual prognosis was poor (0.52 ± 0.34 and 0.22 ± 0.16), OCT images showed retinal atrophy at the fovea despite vitrectomy and sulfur hexafluoride (SF6) gas tamponade.

**Conclusion:**

Early OCT images identified four patterns of contusion maculopathy with different treatment outcomes. In types Ι and II, the visual function and retinal morphology remained intact. With types III and IV, respectively, the treatments of vitrectomy and SF6 gas tamponade for patients were effective.

## Background

About half (54%) of all ocular injuries are minor superficial ocular or periocular injuries. Ocular contusions account for 23%; followed by chemicals and burns (13%), fractures (5%), lid wounds (3%), open globe injuries (2%) and optic nerve injuries (1%) [1]. inadvertent contact with sporting equipment, and tools used during work are the main causes of ocular contusions. Among young people in Japan, sports that include use of a ball; including but not limited to baseball, football, and basketball, are very popular. In these types of sports, blunt traumatic maculopathy resulting from being struck by the balls is the most common mechanism of injury in clinical ophthalmology. However, traumatic macular holes are disproportionally more common in the pediatric and adolescent populations [2]. Treatment success is multifactorial and depends on both ophthalmic factors, e.g., identified diagnostics, treatment methods, timing of surgery, and patient factors, e.g., patient requirements and social barriers.

Optical coherence tomography (OCT) is a relatively new medical diagnostic imaging modality that is being used extensively to observe Retinopathy. By using echo time delay and intensity of backscattered light; OCT can view internal microstructures in biologic tissue with incredible resolution [3, 4]. Coupled with the ability to view multiple cross-sectional images; OCT is a great tool to view macular lesions secondary to macular trauma.

Previous studies [4, 5] using OCT have observed retinal changes in eyes with contusion maculopathy of commotion retinae and traumatic macular hole. Commotio retinae has been known to have good prognosis without interventional therapy. However, there are mixed opinions regarding the benefits of surgical intervention in the treatment of macular holes. In this study, we examine the retinal characteristics of patients diagnosed with contusion maculopathy secondary to sport related ocular trauma using OCT. The question regarding whether surgical or minimally invasive therapy is beneficial will be explored and hopefully better elucidated.

## Methods

In this retrospective study we reviewed the medical records files of twenty-one patients with contusion maculopathy between January 2008 and April 2017 at the Gunma University Hospital. Inclusion criteria included blunt injury and nonpenetrating trauma only. All penetrating trauma were excluded from the study.

All patients underwent a routine ophthalmic examination that included measurement of the best-corrected visual acuity (BCVA), color fundus photography, and OCT examination at multiple time points. We defined 1 week after the ocular injury as the early-period stage. The final examination was defined as that performed after at least 1 month or when the patient refused to undergo examination and treatment. The VA was measured using a decimal chart and converted to the logarithm of the minimum angle of resolution for statistical analysis. When the VA was finger counting, 0.001 was considered the equivalent decimal VA. The data of VA on initial visit were statistically compared with those in final visit with the paired *t* test *P* < .05 was statistically significant.

In our study, the type of OCT system includes SD-OCT (Topcon, Tokyo, Japan), Cirrus high-definition OCT (Carl Zeiss Meditec, Inc., Dublin, CA), and SS-OCT (DRI OCT-1 Atlantis; Topcon, Tokyo, Japan). For OCT examinations, we selected a transverse or vertical display, after generating a macular cube scan (6 × 6 mm area centered on the fovea) and observed changes in the retinal layers of the macula. We evaluated the morphologic changes of retina within the macula during an early stage after injury (within a week after injury) and described the patterns of maculopathy after ocular contusion. The contusion maculopathy was defined as four types: type i: macular commotio retinae, type ii: incomplete macular hole; type iii: macular hole, and type iv: macular hemorrhage. During follow-up, all patients with an ocular contusion received general treatment and/or surgical treatments for macular disorders, which included vitrectomy and sulfur hexafluoride (SF6) gas tamponade.

The study was conducted according to the Declaration of Helsinki. The institutional review board of Gunma University approved the study.

## Results

Twenty-one eyes (11 right eyes, 10 left eyes) of 21 patients (20 males, 1 female) with a macular disorder from blunt ocular trauma were included (Table 1). The ocular contusions resulted from direct injury to the eyeball after being hit by a ball or shuttlecock while playing a sport. Fourteen patients were hit by baseballs, four by footballs, two by shuttlecocks, and one by a tennis ball. The mean patient age at the initial visit was 18.3 years (range, 9–53 years). The mean duration of follow-up was 10.7 months (range, 1–60 months).

**Table 1:** Characteristics of the Subject Population

No	Age^a^	R/L	Equipment	initial VA	Follow period (M)	Final VA	Initial OCT imaging on the fovea	Type	Treatment	Final OCT imaging on the fovea
1	B	L	Tennis	1.2	1	1.2	Outer retinal layer shows thickened and high reflection	I	No	Normal
2	C	R	Baseball	0.8	1	1.2	Vertical high reflective lesion on the fovea	I	No	Normal
3	F	R	Badminton	0.3	11	0.8	EZ irregularity and high reflective lesion on the fovea	I	Lens removal surgery	Normal
4	B	L	Baseball	0.5	3.5	1.2	Macular hole of inner-retinal (V-shaped)	II	No	Normal
5	D	L	Baseball	0.06	1	0.6	Macular hole of inner-retinal (V-shaped)	II	No	EZ disorder
6	C	L	Baseball	0.2	2	0.8	Remaining some retinal tissues on the macular hole-like (V-shaped)	II	No	EZ disorder
7	B	R	Football	0.2	10	1.0	Remaining some retinal tissues on the bottom of hole-like (jar-shaped)	II	No	EZ disorder
8	A	R	Football	0.01	24	0.5	In the center there was a bridge-like retinal tissue	II	No	Retinal atrophy on fovea
9	B	L	Baseball	0.3	8	1.2	In the center there was a bridge-like retinal tissue	II	No	Normal
10	B	R	Baseball	0.1	2.5	0.3	In the center there was a bridge-like retinal tissue	II	No	EZ defect; Retinal atrophy
11	B	L	Baseball	0.6	1	0.7	In the center there was a bridge-like retinal tissue	II	No	Vertical high reflective lesion on the fovea
12	B	R	Baseball	0.001	29	0.8	Full-thickness macular hole and gradually expanded	III	SF6 gas tamponade, vitreous surgery	EZ defect
13	B	R	Baseball	0.2	12	0.9	Full-thickness macular hole and gradually expanded	III	Vitreous surgery	EZ defect
14	B	R	Baseball	0.2	4	0.3	Full-thickness macular hole and gradually expanded	III	No	Full-thickness macular hole
15	C	L	Badminton	0.03	14	0.3	Full-thickness macular hole	III	SF6 gas tamponade vitreous surgery, IOL	Retinal atrophy
16	C	R	Football	0.01	8	0.15	Full-thickness macular hole	III	SF6 gas tamponade, vitreous surgery	^a^Full-thickness macular hole
17	B	R	Football	0.1	20	0.15	Full-thickness macular hole	III	No	Retinal atrophy
18	B	R	Baseball	0.04	6	0.15	Full-thickness macular hole	III	No	EZ defect; retinal atrophy
19	C	L	Baseball	0.01	7	0.7	Submacular hemorrhage, Macular hole?	IV	SF6 gas tamponade	EZ defect; Retinal atrophy
20	B	L	Baseball	0.08	1.5	0.4	Submacular hemorrhage	IV	SF6 gas tamponade	EZ defect
21	D	L	Baseball	0.01	60	0.8	Submacular hemorrhage	IV	Vitrectomy in the fourth year	Full-thickness macular hole

R = right; L = left; VA = visual acuity; M = months; OCT = optical coherence tomography; IOL = intraocular lens; SF_6_ = sulfur hexafluoride; EZ = ellipsoidal zone

Age^a^: providing ages as age-range, A: 1–9, B: 10–19; C: 20–29; D: 30–39; D: 40–49; E: 50–59

**Fig. 1:**
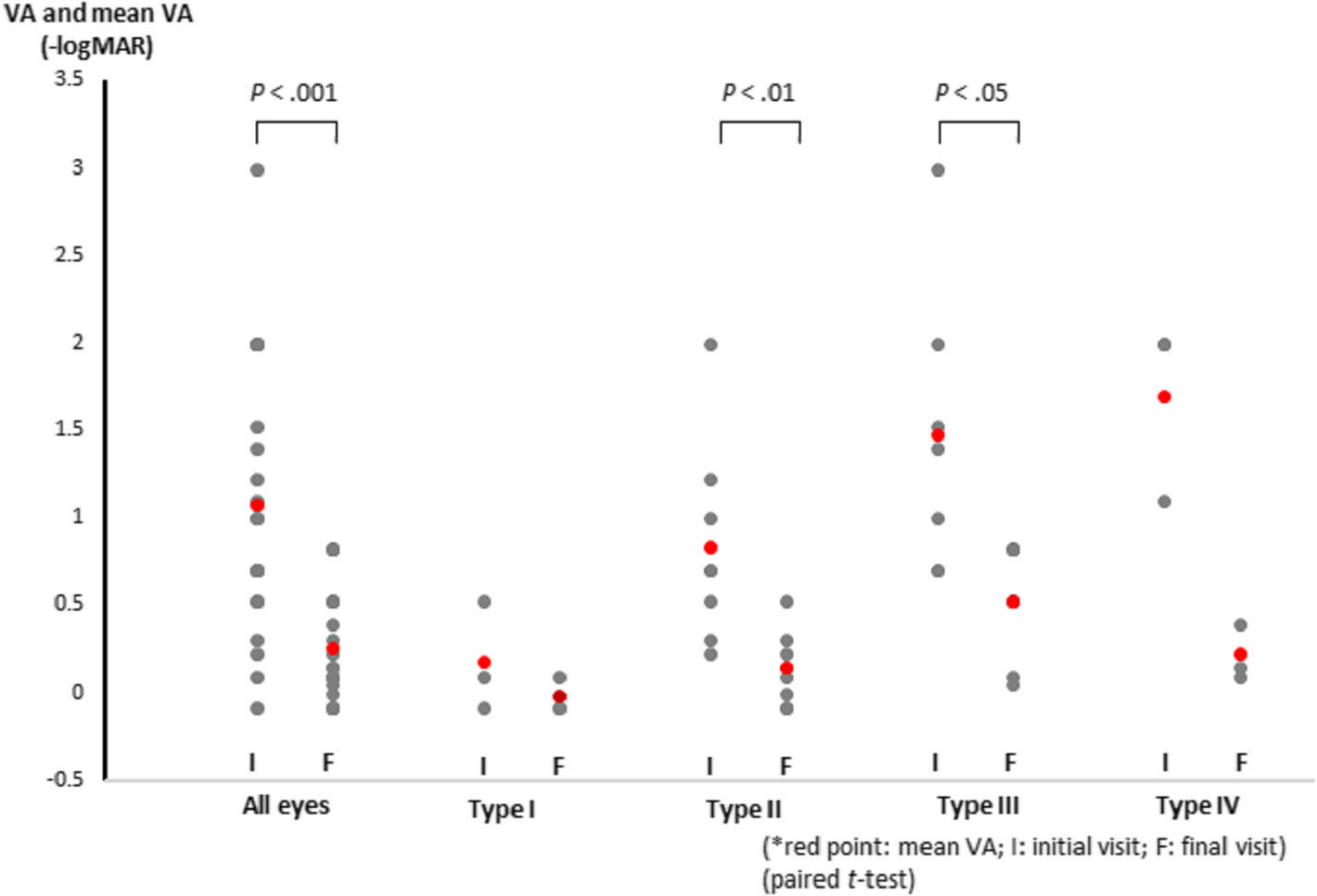
The mean visual acuity (VA) associated with each type of injury during the early and final stages of recovery after contusion maculopathy. logMAR = logarithm of the minimum angle of resolution

At the initial visit, the decimal BCVA ranged from 20 cm/n.d to 1.2 (mean: 1.08 logMAR). The final BCVA were increased (*P* < .001), these ranged from 0.15 to 1.2 (mean: 0.26 logMAR) (Fig. 1). In addition to contusion maculopathy, other findings included dilated pupils (cases 6 and 12), traumatic iris (cases 2, 3, 6,10,14 and 18), hyphema (cases4, 5, 12,15,18, and 21), angle recession (cases 2, 3, 5, and 15), lens subluxation (case 3), traumatic cataract (case 15), and varying degrees of retinal edema or/and hemorrhage outside the macular region (all cases).

### Oct images

OCT was performed at the initial visit -within one week after injury. The Table 1 summarizes all OCT retinal features in all cases. According to the changes in the macular retinal morphology seen on the initial OCT images, the contusion maculopathy was divided into four types: macular commotio retinae, incomplete macular hole; macular hole, and macular hemorrhage.

### Type I

**Fig. 2:**
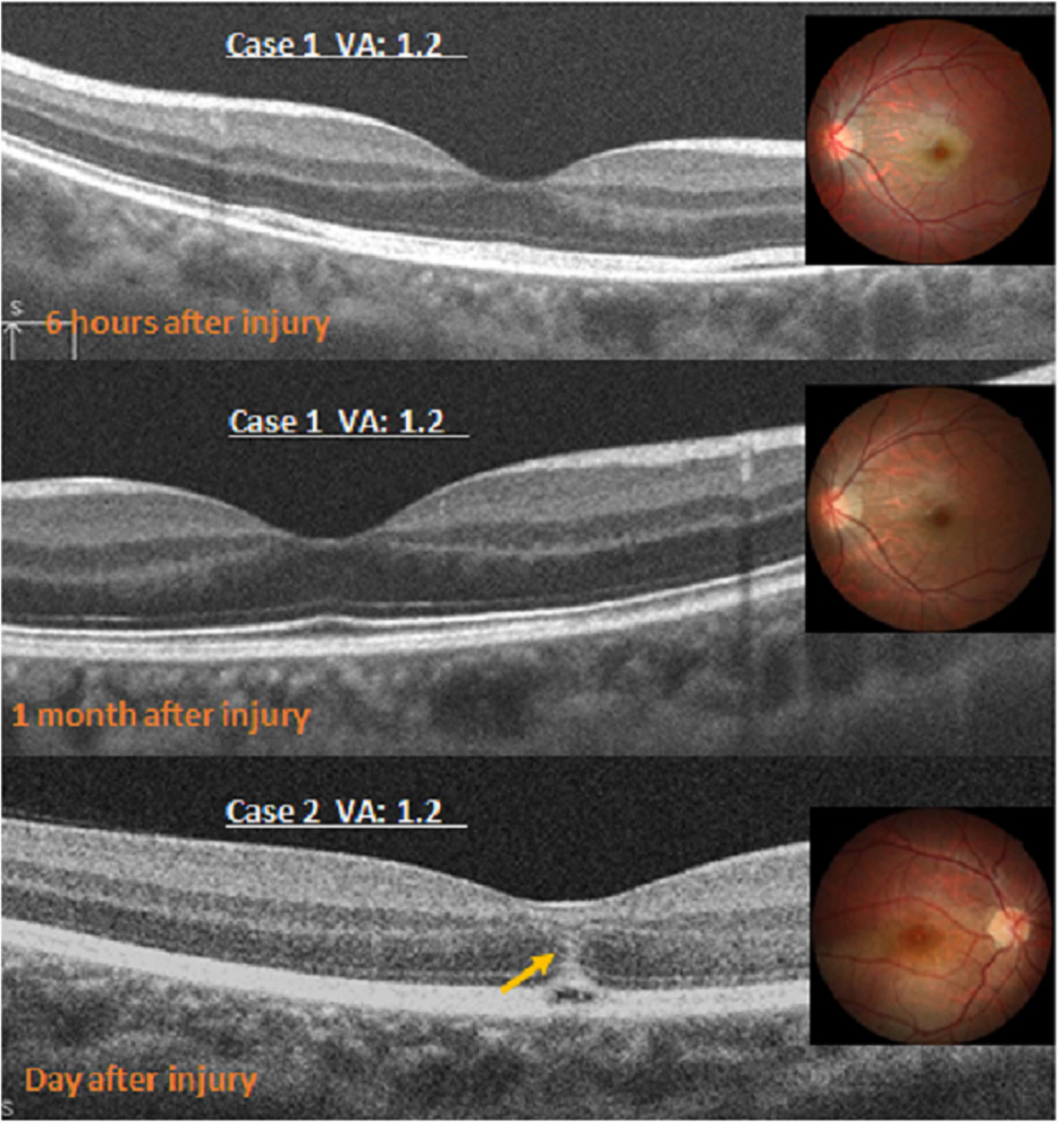
The optical coherence tomography images of a type 1 injury with color photography. Top, Case 1 has abnormal increased reflectivity of the ellipsoid zone (EZ) and retinal pigment epithelium (RPE). Middle, In case 1, the retina is normal after 1 week. Bottom, In case 2, vertical bands of high reflectivity (arrow) on the fovea are seen in addition to the high reflectivity of the EZ and RPE line on the fovea. VA = visual acuity

Commotio retinae was seen on the second day after injury. In cases 1,2, and 3 (14.3%, 3 eyes), the fundus examination showed commotio retinae in the retinal posterior pole. The OCT images showed a merging area of increased reflectivity in the ellipsoidal zone (EZ) and retinal pigment epithelium (RPE) (Fig. 2); a funduscopic photograph showed an opaque white spot on the fovea. After 1 month, a normal retinal structure was seen (Fig. 2). Other OCT changes (Fig. 2) of commotion retinae included a vertical band of high reflectivity with the outer nuclear layer (ONL) on the fovea in addition to the high reflectivity of the EZ and RPE lines. In case 3, during follow up; the OCT showed partial EZ irregularity on the fovea.

In type I, the mean BCVA improved without treatment from the initial level of 0.18 ± 0.31 logMAR (mean ± SD) to a final level of-0.02 ± 0.1 (mean ± SD) logMAR (Fig. 1). In case 3 with traumatic subluxation, lens replacement surgery was performed and the BCVA improved to 0.8 after 11 months. The morphologic structures of the retina on OCT returned to the baseline level in the three cases, after 1 month (case 1 and 2), and 11 months (case 11).

### Type II

**Fig. 3:**
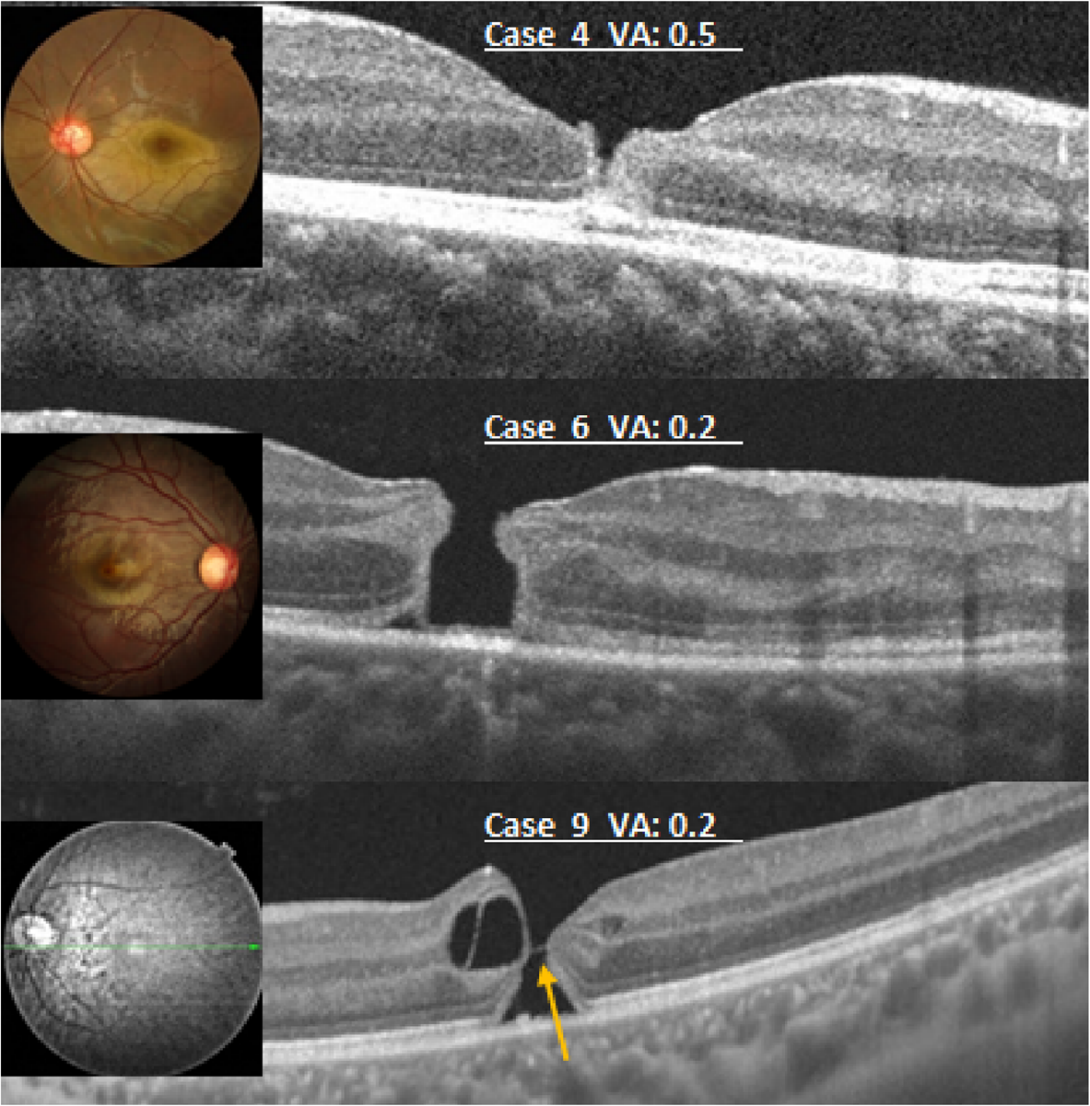
Theoptical coherence tomography images of a type 2 injury with color photography. Top, The image shows a V-shaped separation of the inner retinal layer at the fovea. The bottom of the hole-like has a complete ellipsoidal zone (EZ). Middle, The image shows an incomplete jar-shaped macular hole with a complete EZ. Bottom, A bridge (arrow) is seen connected to the retinal tissues in the middle of a macular hole-like structure. VA = visual acuity.

OCT showed an incomplete macular hole in cases 4 to 11 (38.1%, 8 eyes) at the initial visit (Fig. 3) that we defined as type II contusion maculopathy. The OCT images of cases 4,5, and 6 (Fig. 3) appear as separation of the inner retinal layer at the fovea with a complete EZ. As seen in case 4; a V-shaped macular hole in an 11-year-old boy, had an initial BCVA of 0.5 in the left eye immediately after he sustained the ocular trauma. 3.5 months later, the BCVA recovered to 1.2 without treatment. OCT images of the fovea showed a normal retina. In cases 5 and 6, after 2 weeks and 2 months, respectively, without surgical treatment, OCT showed normal macular concavity with a disrupted EZ. The BCVAs recovered from the initial levels of 0.06 and 0.2 respectively, to the final levels of 0.2 and 0.8.

Case 7 appeared to be a macular hole (Fig. 3); however, in the inferior fovea, an irregular layer of outer retinal tissue with EZ contributed to a skewed contour, which we described as “jar-like”. After 10 months without surgical treatment, OCT showed the retina had a slight EZ defect. The BCVA increased from the initial level of 0.2 to1.0.

The OCT feature in cases 8 to 11 demonstrated a bridge connected to the retinal tissue in the middle of the macular holes (Fig. 3). The bridge appeared at different levels in the macular holes. Without surgical treatment, the OCT images in case 9 were normal, and the other eyes experienced some recovery. However, each case showed varying degrees of EZ defect and macular dystrophy.

During follow-up, in these eyes with type II, no retinal detachments occurred around the macular holes, and these hole-like defects tended to close; thus, we did not perform surgery. The mean BCVA (Fig. 1) increased from the initial level of 0.83 ± 0.57logMAR (mean ± SD) to 0.14 ± 0.21 logMAR (*P* < .01). The final OCT images of the fovea of patient 8 and 10 showed retinal dystrophy with a final BCVA of 0.5 and 0.3, the other eyes showed a normal retina or EZ defect with better final vision.

### Type III

**Fig. 4:**
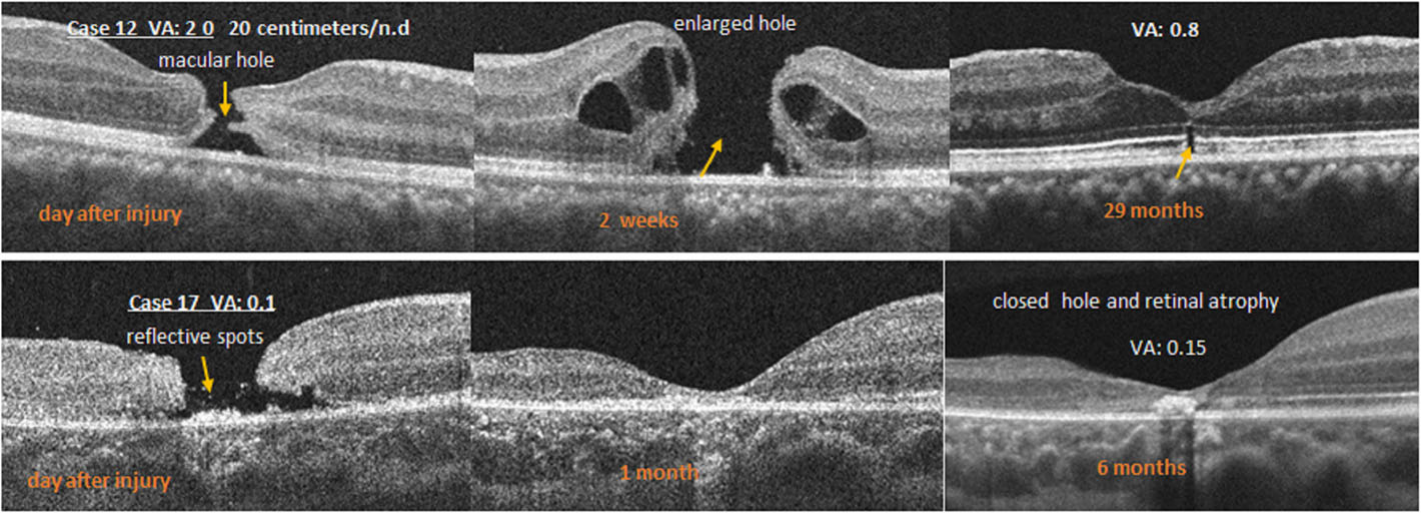
Top, An optical coherence tomography (OCT) image of case 12 shows a full-thickness macular hole on the left. After 2 weeks, the hole expanded gradually with a retinal detachment around the hole (middle). The final OCT image shows that the hole closed with a defect of the ellipsoidal zone (EZ) defect at the fovea (right). Middle, The left OCT image in case 17 shows a full-thickness macular hole 2 days after the injury and some reflective spots in the middle of the hole (arrow). The middle OCT image shows that the hole closed 1 month after the injury. After 6 months, the hole is closed and an atrophic scar is seen on the fovea (right)

OCT showed full-thickness macular holes in cases 12 to 18 (33.3%, 7 eyes) during the early stage after injury (Fig. 4). The mean BCVA in type III increased from the initial level of 1.47 logMAR to 0.52logMAR (*P* < .05) (Fig. 1). Figure 4a shows a full-thickness macular hole that was not surrounded by a localized retinal detachment on the day after the injury (case 12). During follow-up, the hole expanded gradually, and a retinal detachment developed gradually around the hole. On the third week, 0.3 ml of pure SF6 was injected into the vitreous cavity; however, the macular hole did not close postoperatively. Two weeks later, we repaired the macular hole during vitrectomy. The final OCT image showed a localized EZ on the fovea, and the BCVA recovered to 0.8. The macular holes in cases 13 and 14 also enlarged gradually similarly to case 12. The final BCVA in case 13 reached 0.9 after vitrectomy. In case 14, the patient refused surgery and 3 months after the injury, the macular hole remained open and the final BCVA was only 0.3.

In cases 15 and 16, same as case 12, after pure SF6 was injected into the vitreous cavity, the holes remained open, but they closed after vitrectomy. The final OCT image showed macular retinal dystrophy in cases 15. Case 16 had recurrence of macular hole, and he terminated his treatment. The final BCVAs were 0.3 and 0.15, respectively.

In cases 17 and 18, some spots of reflectivity of varying sizes were seen within the macular holes on OCT (Fig. 4). The holes were gradually reduced during follow-up visits. Although no surgery was performed, the macular holes closed after1 month. The final OCT images showed an extremely thin retina at the fovea and macular dystrophy, and the BCVAs were poor at 0.15.

### Type IV

**Fig. 5:**
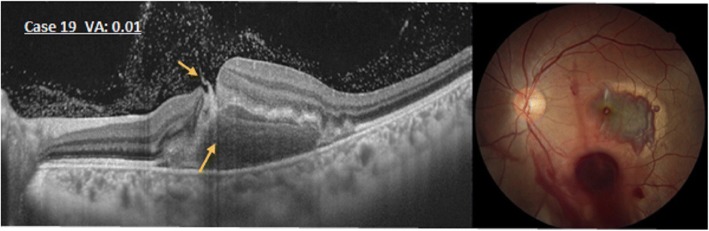
An optical coherence tomography image of the eye in case 19 shows that the retina is elevated. Some blood (arrows) is seen between the separated fovea, on the retinal surface, and the subretinal hemorrhage

Macular hemorrhages developed during the early stage after injury in cases 19, 20, and 21 (14.3%, 3 eyes). The OCT images showed elevation of the retina (Fig. 5), as well as a completed RPE line and Bruch’s membrane. Some blood was visible between the separated foveal pit, on the retinal surface, and in the vitreous cavity. Cases 19 and 20 were treated with pure SF6 on the thirteenth and seventeenth day respectively after injury. One month later, OCT images showed a deeper fovea with EZ defect and a thinning retina in case 19. Comparatively; case 20 only had an EZ defect. The blunt traumatic maculopathy observed in case 21 was not treated surgically; it was characterized by an emerging macular pseudo-hole 1 year after the injury. Four years after the injury, a full-thickness macular hole was seen, and vitrectomy was performed. In all three cases, their mean BCVA from initial 1.7 logMAR (0.01, 0.08, 0.01, respectively) increased to 0.22 logMAR (0.7, 0.4, 0.8, respectively).

## Discussion

In the current retrospective study, we evaluated the OCT images of contusion maculopathy in 21 patients. Blunt sports-related trauma resulting from balls and shuttlecocks hitting the eyes were the main mechanisms of injuries. Based on the OCT images obtained during the early stage of ocular injury (within 1 week), we divided the contusion maculopathy into four types: Ι, commotio retinae (14.3%); II, incomplete macular holes (38.1%); III, macular holes (33.3%); and IV, macular hemorrhage (14.3%). The proportion of macular holes with incomplete (type III) and full-thickness (type IV) contusion maculopathy are larger, about 71% (38.3% + 33.3%). Our results reveal differing treatment approaches which alter prognosis in the two types of contusion maculopathy. Based on these results, it is important to distinguish these two forms of contusion maculopathy by OCT features.

The term commotio retinae describes the damage to the outer retinal layers caused by shock waves that traverse the eye from the site of impact after blunt trauma. Using OCT, Itakura and Kishi [5] reported acute abnormalities of the EZ in commotio retina and restoration of the photoreceptor architecture accompanied by improvement in BCVA. In the three patients with a type Ι injury, the OCTs on the initial visit showed not only thickened and increased reflectivity of the EZ and RPE but also injury in the outer nuclear layer (ONL) (Fig. 2). Although this has been attributed to extreme paretic vasodilatation, it is more likely caused by a change in the transparency of the intracellular proteins of the retina. Change in transparency of retina likely results as a direct response to either contusion or brief ischemia secondary to injury of choroidal or retinal blood vessels. During the early stages in a type Ι injury, we did not find EZ defects. However, during the retinal recovery stage, we noticed an evanescent EZ defect phase. This phase might be considered as retinal edema in the early stages, eventually leading to small amounts of photoreceptor cell damage. Depending on the degree of damage, the times to complete recovery varied from 1 month to 11 months. In the end their BCVA recovered to normal 1.0.

In routine fundus examination, the eyes suspected of macular holes were diagnosed as incomplete macular holes after detailed OCT analysis. Based on the OCT images, 38.1% of all cases were classified with incomplete macular holes (type II injury). Their images included the following three retinal changes: 1) AV-shaped macular hole, 2) Jar shaped macular hole, and 3) Bridge shaped macular hole. The AV-shaped appearance was characterized by a separation between the retinal surface and the ONL; the outer retinal tissues of the external limiting membrane and EZ layers remained at the bottom of the hole (Fig. 3). The jar-shaped appearance was characterized by the EZ on the wide bottom of the macular hole (Fig. 3). The bridge-shaped appearance was characterized by a bridge connected to retinal tissue in the middle of the full-thickness macular hole (Fig. 3). As a result, residual macular retinal atrophy was observed. The eyes with type II injury had better BCVA (mean ± SD, 0.14 ± 0.21 logMAR) compared to type III (mean ± SD, 0.52 ± 0.34 logMAR), and had natural closing of the macular hole. It can be inferred OCT examination of eyes with contusion maculopathy can directly assess the prognosis of the patients.

In this study, we found that the cases with three retinal changes in type II injury were significantly correlated with visual and retinal morphologic improvements. In addition, no cases with a type II injury progressed to full-thickness macular holes (type III), despite appearance of residual retinal EZ defect and atrophy at the fovea. These changes are similarly observed in idiopathic lamellar macular holes. Some researchers have reported that lamellar macular holes usually remain stable over time, with few eyes evolving into full-thickness macular holes, and some cases resolve spontaneously [6–8].

We observed that the early OCT images of traumatic macular holes (type III) did not show localized retinal detachments around the macular holes, but the holes expanded gradually. This differed from idiopathic macular holes [9, 10], which are thought to result from focal shrinkage of the vitreous cortex in the foveal area. In contrast, traumatic macular holes result from traumatic shock. The results in the current cases indicated that SF6 gas tamponade was ineffective, while vitrectomy was the more effective treatment method as with idiopathic macular holes [11–14]. The current results indicated that although surgery can be successful, once the macular holes have formed, it is easy for macular atrophy to occur, and recovery of visual function is not good. The final mean BCVA is less than 0.3 (mean ± SD, 0.52 ± 0.34 logMAR), while in eye with type II injury is more than 0.5 (mean ± SD, 0.14 ± 0.21 logMAR). Even if our number of cases is relatively small, we still think that if there are punctate high reflective tissues in the macular hole, or the hole has no tendency to expand, it may eventually close naturally (case 17 and 18), otherwise surgical intervention is recommended.

A high spontaneous closure rate was observed, with a trend toward smaller OCT dimensions. Surgical intervention was less successful at hole closure when elected after 3 months [2]. In our study cases, all eyes of Type II injury had successful spontaneous hole closure as compared to type III where vitrectomy was required. Therefore, it is very important to distinguish incomplete and full-thickness macular hole by OCT features at the early stage of trauma.

Bruch’s membrane is an elastin- and collagen-rich extracellular matrix that acts as a molecular sieve. Blunt trauma of the eye can cause Bruch’s membrane tear and choroidal rupture, resulting in vitreous and subretinal bleeding. In our type IV cases, Bruch’s membranes were complete on OCT. This suggests subfoveal and vitreous hemorrhages may originate from retinal vessels excluding ruptures. In the early phase of injury, due to retinal or vitreous hemorrhage in the fovea/macula; it was difficult to determine if macular holes were present. Type IV contusion maculopathy was defined once a definite macular hole was established. One treatment with SF6 gas tamponade for eyes with type IV can promote absorption of bleeding and early retinal anatomic recovery. In cases 19 and 20, the result of our treatment was effective. Ocular trauma in younger patients demonstrate proliferation of vitreous cortex after absorption of macular hemorrhage. This may be in part facilitated by the presence of a macular hole [12, 14–16]. Case 21 is an example of this. Long-term clinical follow-up of traumatic maculopathy after ocular contusion is important. OCT becomes an important imaging modality in differentiating types of contusion maculopathy. As seen in the cases presented in our study, treatment outcomes and prognosis are directly related to type of OCT imaging seen during follow-up.

## Conclusion

The current retrospective study indicated that the changes associated with contusion maculopathy in the early stage (within 1 week) after blunt injury can be divided to four patterns with different outcomes: type 1, macular commotio retinae; type II, incomplete macular hole; type III, macular hole; and type IV macular hemorrhage. Eyes with types Ι and II can achieve better visual outcomes without surgery (mean ± SD, logMAR. 0.02 ± 0.1 and 0.14 ± 0.21). With types III and IV, a more invasive approach involving vitrectomy and SF6 gas tamponade was required for adequate response in results. OCT imaging is effective in differentiating Type I/II from Type III/IV contusion maculopathy, ultimately altering treatment decision and overall prognosis for patients.

## Abbreviations

BCVA: Best-corrected visual acuity
EZ: Ellipsoidal zone
OCT: Optical coherence tomography
ONL: The outer nuclear layer
RPE: Retinal pigment epithelium
